# An Amyloidogenic Sequence at the N-Terminus of the Androgen Receptor Impacts Polyglutamine Aggregation

**DOI:** 10.3390/biom7020044

**Published:** 2017-06-19

**Authors:** Emmanuel Oppong, Gunter Stier, Miriam Gaal, Rebecca Seeger, Melanie Stoeck, Marc-André Delsuc, Andrew C. B. Cato, Bruno Kieffer

**Affiliations:** 1 Department of Integrative Structural Biology, Institut de Génétique et de Biologie Moléculaire et Cellulaire, INSERM, U964, CNRS, UMR-7104, Université de Strasbourg, 1 rue Laurent Fries, 67404 Illkirch–Graffenstaden, France; emmanopp@yahoo.com (E.O.); Delsuc@igbmc.fr (M.-A.D.); 2 Heidelberg University Biochemistry Center (BZH), INF 328, D-69120 Heidelberg, Germany; gunter.stier@bzh.uni-heidelberg.de; 3 Institute of Toxicology and Genetics, Karlsruhe Institute of Technology, Hermann-von-Helmholtz-Platz 1, 76344 Eggenstein-Leopoldshafen, Germany; miriamgaal@icloud.com (M.G.); rebecca.seeger@kit.edu (R.S.); 4 Institute for Photon Science and Synchrotron Radiation, Karlsruhe Institute of Technology, Hermann-von-Helmholtz-Platz 1, 76344 Eggenstein-Leopoldshafen, Germany; melanie.stoeck@gmx.de

**Keywords:** amyloid peptides, androgen receptor, nuclear receptor, aggregation, atomic force microscopy

## Abstract

The human androgen receptor (AR) is a ligand inducible transcription factor that harbors an amino terminal domain (AR-NTD) with a ligand-independent activation function. AR-NTD is intrinsically disordered and displays aggregation properties conferred by the presence of a poly-glutamine (polyQ) sequence. The length of the polyQ sequence as well as its adjacent sequence motifs modulate this aggregation property. AR-NTD also contains a conserved KELCKAVSVSM sequence motif that displays an intrinsic property to form amyloid fibrils under mild oxidative conditions. As peptide sequences with intrinsic oligomerization properties are reported to have an impact on the aggregation of polyQ tracts, we determined the effect of the KELCKAVSVSM on the polyQ stretch in the context of the AR-NTD using atomic force microscopy (AFM). Here, we present evidence for a crosstalk between the amyloidogenic properties of the KELCKAVSVSM motif and the polyQ stretch at the AR-NTD.

## 1. Introduction

The human androgen receptor (AR) is a ligand inducible transcription factor and a member of the nuclear receptor family that includes the glucocorticoid receptor (GR), mineralocorticoid receptor (MR), estrogen receptor (ER), and progesterone receptor (PR). This protein consists of 919 amino acids and is organized into an amino terminal domain (NTD, 1–559), a DNA binding domain (DBD, 559–624), a hinge region (HR, 624–706), and a carboxy-terminal ligand-binding domain (LBD, 706–919). The LBD and DBD display conserved and well characterized three-dimensional folds [1,2,3]. In contrast, the NTD is described as an intrinsically disordered region, a feature that hampers the elucidation of simple sequence-function relationship [4]. Notwithstanding its lack of a defined fold, several features of the transcriptional activity of AR are attributed to the NTD where several short peptide motifs are involved in the modulation of full-length AR transcriptional activity [5,6]. AR-NTD sequence also features several low complexity regions, such as homopolymer stretches of glutamines (polyQ), glycines (polyG), and prolines (polyP), whose biological role remains unknown [7]. Extension of the polyQ sequence above 40 residues is, however, associated with spinal and bulbar muscular atrophy (SBMA), a neurodegenerative disease [8], while a shortening of this homopolymer below 20 residues may be responsible for increased prostate cancer risk [9]. A correlation between the length of the polyQ sequence, the morphology of AR oligomers and AR-mediated neurotoxicity has been established [10]. Atomic force microscopy (AFM) analyses of full-length AR showed that AR with 65 glutamine residues (ARQ65) forms fibrillar oligomers, which are toxic in neuronal cells while AR with 22 glutamines forms non-toxic annular oligomers [10].

Protein oligomerization mediated by polyQ stretches are often modulated by the presence of adjacent domains or short sequence motifs that can either act as enhancers or repressors of the oligomerization process [11]. Recently, biophysical studies showed that a leucine-rich motif located at the N-terminal edge of AR polyQ region has an inhibitory effect on polyQ-mediated AR aggregation [12]. Other examples where the aggregation of a polyQ stretch is modulated by its flanking regions can be found in Huntington, Ataxin 3, and Ataxin 1, where the 17 amino acids N-terminal sequence of Huntington, the Josephin domain (JD) and the AXH domain trigger a first step of aggregation of polyQ tracts containing proteins [13,14,15]. Although these sequences are non-polyQ, they have an intrinsic property to aggregate, and this impacts on the aggregation properties of their adjacent polyQ counterparts.

We have recently shown that synthetic KELCKAVSVSM peptides derived from the AR-NTD has an intrinsic property to form amyloid fibril under mild oxidative conditions [16]. It is therefore likely that this sequence would have an effect on aggregation of the polyQ track at the AR-NTD. In the present communication, we present evidence for a crosstalk between the amyloidogenic properties of the KELCKAVSVSM motif and the polyQ stretch at the AR-NTD.

## 2. Results

### 2.1. Amyloidogenic Properties of KELCKAVSVSM Peptides Expressed as SUMO Fusion Proteins

**Figure 1:**
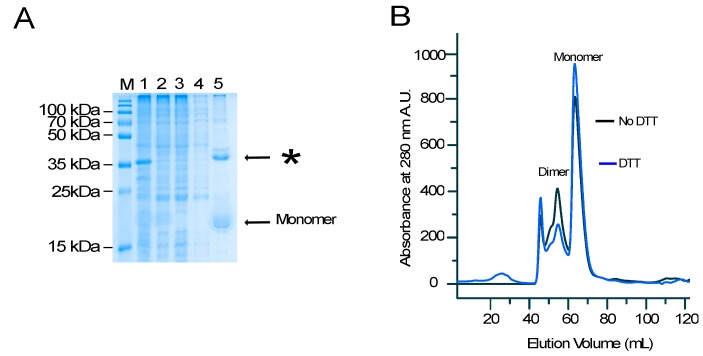
(**A**) Non-reducing sodium dodecyl sulfate polyacrylamide gel electrophoresis (SDS-PAGE) analysis of an affinity-purified poly Histidine-Small Ubiquitine-like Modifier protein (His-SUMO) fusion expressed in *Escherichia coli* (*E. coli)* showing the total cellular extract (Lane 1), the soluble cell extract (Lane 2), the flow-through fraction from the nickel column (Lane 3), the wash fraction (Lane 4), and the eluted fraction (Lane 5). M: molecular weight. (**B**) Size exclusion chromatography of the HisSUMO peptide in buffer with dithiothreitol (DTT) (blue line) and without DTT (black line).

We have previously reported amyloidogenic properties of a conserved sequence from the central region of AR-NTD [16]. Experiments with peptides of different lengths identified the sequence KELCKAVSVSM as the minimal motif for the formation of amyloid fibers upon the addition of 10% dimethyl sulfoxide (DMSO). To determine whether the amyloidogenic property of this sequence is retained in the context of a fusion protein, we fused the peptide to a Small Ubiquitine-like Modifier protein SUMO and expressed it in *Escherichia coli* (*E. coli*) [17]. After affinity purification of the bacterial cell lysates on nickel agarose resins, a major product with the expected mass of the His-SUMO–peptide fusion protein of 15,819 kDa was identified on a sodium dodecyl sulfate polyacrylamide gel electrophoresis (SDS-PAGE) (Figure 1A, Lane 5). In addition, a distinct protein with a higher molecular weight was also identified (Figure 1A, see asterisks).

To determine the identity of the additional product, the HisSUMO–peptide fusion was subjected to size exclusion chromatography (SEC) with or without the addition of the reducing agent, dithiothreitol (DTT) (Figure 1B). Both conditions led to similar SEC profiles with a major sharp and symmetrical peak eluting at 64 mL, indicative of a pure and homogenous protein. Another peak, eluting from 55 to 60 mL was also identified, although this fraction was significantly decreased when DTT was added to the elution buffer (Figure 1B). Mass spectrometry analysis unambiguously identified the lower and higher molecular weight fractions to be SUMO-KELCKAVSVSM fusion protein and its covalent dimer, respectively. Since SUMO has no cysteine in its sequence, the observed dimerization may be attributed to the formation of an inter-molecular disulfide bridge mediated by the cysteine within the peptide.

**Figure 2:**
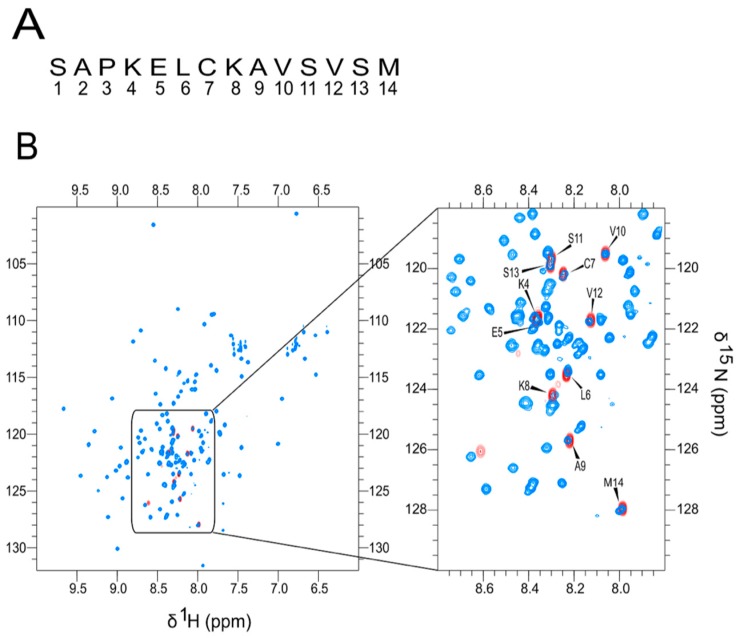
^1^H-^15^N Heteronuclear Single Quantum Correlation (HSQC) spectrum of KELCKAVSVSM (**A**) The peptide sequence. (**B**) An overlay of the amide region of the ^1^H-^15^N HSQC spectrum of the HisSUMO–peptide fusion (blue) and the cleaved peptide (red) and to the left a blow-up of the relevant region of the HSQC spectrum.

We have previously shown that the KELCKAVSVSM forms fibrils under mild oxidation conditions [14]. We therefore determined whether the SUMO-KELCKAVSVSM fusion proteins (either in the monomer or dimer form) would form amyloid fibers in the presence or absence of DMSO, but this failed after repeated attempts. Since the SUMO tag may mask the formation of the fibrils, we determined the accessibility of the peptide in the context of the fusion protein. We compared the ^1^H-^15^N heteronuclear single quantum coherence (HQSC) spectra with or without the SUMO tag to determine the disordered state of the peptide within the fusion protein. The ^1^H-^15^N HQSC correlations corresponding to the peptide alone (without the tag) was completely superimposable on the correlations present in the HisSUMO–peptide fusion in the spectral region corresponding to amide protons from disordered residues (8.0 to 8.4 ppm, see Figure 2). This finding highlights the lack of interactions between the SUMO fusion protein and the peptide, suggesting that the inhibition of the fibrillation process in the SUMO peptide fusion cannot be attributed to a lack of peptide accessibility.

**Figure 3:**
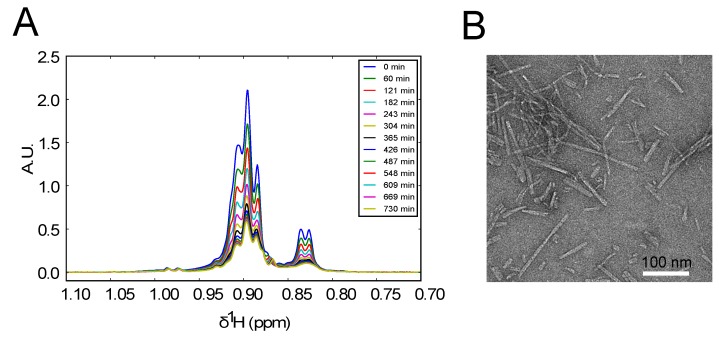
(**A**) Time-dependent proton NMR spectra of the methyl region of the KELCKAVSVSM peptide recorded shortly after addition of 10% dimethyl sulfoxide (DMSO) at 25 °C. The observed decrease of signal intensity is due to the formation of amyloid fibers that are not observed in the spectrum. (**B**) A Transmission Electronic Microscopy (TEM) image of the NMR sample 24 h after the addition of 10% DMSO showing the presence of fibrils of variable length.

We then cleaved the SUMO tag, HPLC purified the cleaved peptide and added 10% DMSO to the sample. DMSO is a mild but specific oxidant of cysteines [18] that triggers the formation of covalent dimers, which could be followed by the self-association of the dimers into amyloid fibers. Proton 1D NMR spectra recorded every half hour for 24 h after the DMSO addition showed the gradual time-dependent disappearance of all the resonances corresponding to the formation of large sedimenting oligomers indicative of amyloid fibril formation (Figure 3A). This was confirmed by transmission electron microscopy (TEM) analysis of the pelleted material in the NMR tube after the 24 h incubation. The resulting images displayed numerous fibrils with a large distribution of lengths but similar widths, in agreement with our previous report [14].

### 2.2. The KELCKAVSVSM Sequence Modulates polyQ Oligomerization Properties of AR-NTD Fragments

**Figure 4:**
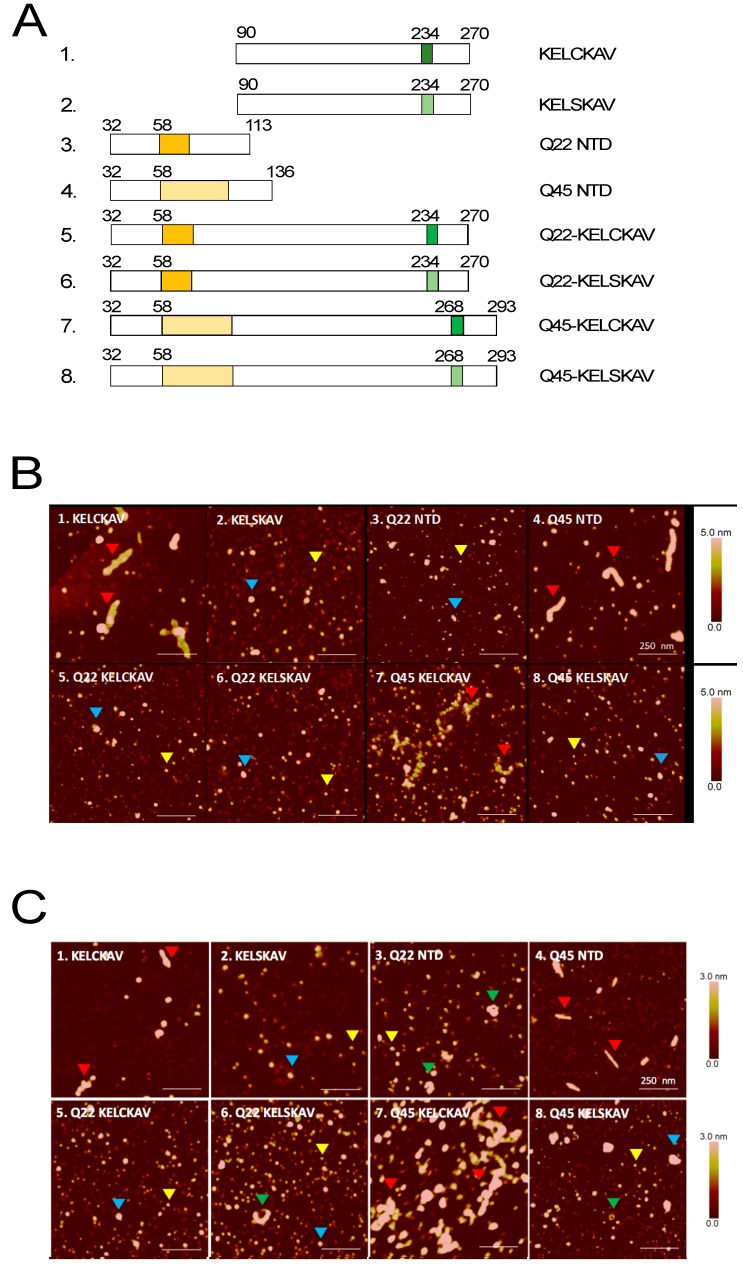
Morphology of Androgen Receptor-Amino-Terminal Domain (AR-NTD) oligomers. (**A**) Schematic representation of amino terminal domain (AR-NTD) peptides covering the polyQ stretch (Q22 and Q45) stretch and the KELCKAVSVSM sequence (in dark green) or with the cysteine mutation KEL**S**KAVSVSM (light green). (**B**) Glutathione S-Transferase (GST) tagged AR-NTD or (**C**) untagged AR-NTD proteins were incubated for 18 h at 37 °C to initiate the aggregation process before spotting on mica for atomic force microscopy (AFM) measurements. Blue and yellow arrowheads refer to smaller and larger globular oligomers (SGOs and LGOs); green arrowheads refer to annular oligomers and red arrow heads to fibrillar oligomers.

As peptide sequences with intrinsic ability to oligomerize are reported to have an impact on the aggregation of polyQ tracts [19], we determined the effect of the KELCKAVSVSM on the polyQ stretch in the context of the AR-NTD. We expressed the KELCKAVSVSM (with or without a cysteine to serine mutation) and polyQ stretches of 22 or 45. As controls, we also expressed sequences covering only the KELCKAVSVSM with or without the cysteine mutation or only polyQ stretches of 22 or 45 (Figure 4A). All the peptides were first expressed as GST fusion proteins, and their conformation was determined by AFM.

These analyses revealed the formation of smaller and larger globular oligomers (SGOs and LGOs) with diameters of 9–24 and 35–68 nm, as we have previously published [10] (Figure 4B; yellow and blue arrowheads). Fragments containing either an expansion of the polyQ stretch to 45 (Q45) or the KELCKAVSVSM sequence formed fibrillar oligomers even in the absence of DMSO (Figure 4B, red arrowheads Panels 1, 4), and fibrils were also observed when both sequences were present (Panel 7). However, fibrillar oligomers were undetectable when the cysteine residue was changed into a serine (Figure 4 A,B; Panels 2 and 8).

To rule out a possible effect of the GST tag on the fibrillization process, we re-cloned the DNA sequences coding for these peptides into a pET-Gb1a vector and cleaved off the GB1 tag with a TEV protease after protein production to release untagged AR-NTD fragments. Analysis of these untagged fragments by AFM produced results similar to those obtained with the tagged peptides (compare Figure 4C with Figure 4B). The only difference was a slight variation in the size and shape of the fibrillar oligomers. The fibrillar oligomers in the GST tagged fragments were thicker than their untagged counterparts and annular structures were additionally detected particularly in samples that contain the untagged Q22. (Figure 4C; Panels 3 and 6; arrowheads). The fibrillar oligomers of the untagged peptide also showed some differences. The untagged Q45 fibrils were thinner and shorter (width 28 ± 13 nm; length 183 ± 95 nm) compared to the untagged KELCKAVSVSM fibrils (width 60 ± 15 nm; length 293 ± 102 nm). The Q45-KELCKAV fibrils formed by the fragment containing both the polyQ amplification and the KELCKAVSVSM were very different. They consisted of bundles of fibrillar aggregates that were longer and thicker than those formed by either the Q45 or the KELCKAVSVSM peptides (Figure 4B,C; Panel 7). These aggregates were no longer visible when the cysteine in KELCKAVSVSM was substituted by a serine (Figure 4B,C; Panel 8). No significant change in the structure of oligomers was observed in fragments containing Q22 and KELCKAVSVSM with or without a cysteine to serine mutation (Figure 4B,C; Panels 5 and 6). Thus, the major changes in morphology of the oligomers following the cysteine to serine exchange in the KELCKAVSVSM sequence were detected in fibrillar oligomers formed by the KELCKAVSVSM itself or the Q45-KELCKAVSVSM but not the Q22-KELCKAVSVSM sequence. This suggests that the KELCKAVSVSM motif contributes to fibril formation of the larger fragments containing amplified polyQ (poly 45).

## 3. Discussion

There are nine proteins that contain polyQ stretches, which when amplified promote protein aggregation and are neurotoxic. This phenomenon has generated a family of polyQ disorders such as Huntington’s disease, several spinocerebellar ataxias or the X-linked spinal and bulbar muscular atrophy (SMBA) [20]. The aggregation properties of the polyQ sequences of these proteins is controlled by many factors among which are intrinsic factors such as their length but also the presence of adjacent sequence motifs [19].

The role of flanking sequence motifs as modulators of polyQ-mediated protein aggregation is currently emerging as a common mechanism for aggregation. So far there are three well-known examples of this. A 17 amino acid N-terminal domain (N17) that flank the polyQ tract of huntingtin is known to have self-association properties that promote polyQ aggregation of Huntington [21,22]. The JD of Ataxin 3 displays a fibrillogenic behavior that affects the aggregation of Ataxin 3 [14], and a further example is the ataxin-1/HBP1 (AXH) domain of ataxin-1 that also controls polyQ-mediated aggregation of Ataxin 1 [15]. So far, no such domain has been identified in the remaining six out of the nine polyQ proteins. We have previously identified an evolutionary conserved sequence motif KELCKAVSVSM at the N-terminus of the AR that formed fibrillar aggregates but not when the cysteine that it contains was converted into a serine [16]. The aggregates formed by this sequence bound thioflavin T, which is a feature shared by amyloid fibers and polyQ aggregates, although the sequence itself is non-polyQ. Previous in vitro studies have shown that the KELCKAVSVSM peptide forms fibrils under mild oxidative conditions [16]. Other studies have reported that it has an intermediate helical structure, even under non-oxidative conditions [23], and that mutation of this sequence impairs wild-type AR response [24]. A possibility therefore exists that the inherent aggregation property described for this sequence could function as a nucleation center in the aggregation of the polyQ stretch 200 amino acids upstream and impair AR action.

**Figure 5:**
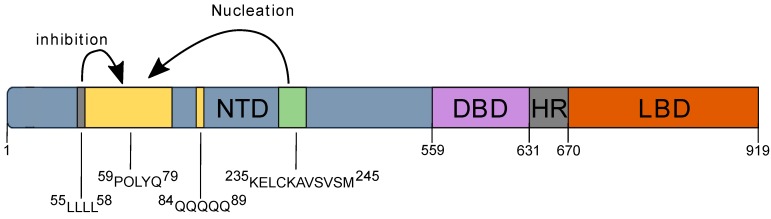
Schematic view of the different possible interactions in androgen receptor (AR) with an impact on AR aggregation. NTD: N-Terminal Domain; DBD: DNA Binding Domain; HR: Hinge Region; LBD: Ligand Binding Domain.

In the present report, we show that the aggregation property of the KELCKAVSVSM peptide is heavily impacted by its neighboring sequences. When tagged with a SUMO protein, the KELCKAVSVSM peptide formed only dimers and no further high molecular weight species. When the SUMO tag was cleaved, the peptide underwent further aggregation to form fibrils. However, when the length of the peptide was extended by over 200 amino acids to encompass the expanded polyQ stretch (Q45) of the AR, it potentiated the inherent property of the amplified polyQ stretch (Q45) to form fibrils. This shows a positive influence of the KELCKAVSVSM peptide on polyQ stretch fibrillization at the AR-NTD. A leucine-rich sequence located at the N-terminal edge of the polyQ region has been shown to have an inhibitory effect on polyQ-mediated AR aggregation [12]. It therefore appears that an interplay of positive and negative *cis*-regulatory elements controls the aggregation properties of the polyQ-containing proteins of which the KELCKAVSVSM motif is the most important player, as depicted in Figure 5.

We could also show in our study that, when the polyQ stretch in the surrounding AR sequences of the KELCKAVSVSM is 22 rather than 45, no fibrillar oligomers are formed. It is therefore important to note that not only are the KELCKAVSVSM motif, the polyQ stretch, and the primary amino acid composition important in determining the state of aggregation at the AR-NTD, but the distance between the KELCKAVSVSM motif and the polyQ stretch is as well. While our study shows the contribution of *cis*-regulatory elements to the oligomerization of the AR-NTD, *trans*-acting factors could play an equally important role. The KELCKAVSVSM sequence is reported to be a binding site for the cochaperone, C-terminal heat shock 70 interacting protein (CHIP) [25], and to partially overlap with the binding site for the RNA polymerase associated protein 74 subunit of the general transcription factor TFIIF [24]. Moreover, a recent study reported on the intrinsic ability of AR-NTD sequences expressed as a decoy molecule to reduce the expression of the androgen-regulated genes, [26]. How all these factors contribute to the overall structure of the AR-NTD remains to be established. Nonetheless our present results on the KELCKAVSVSM motif and the polyQ stretch of the AR clearly demonstrate the existence of a crosstalk between these two *cis*-regulatory elements in the control of polyQ stretch aggregation.

## 4. Materials and Methods

### 4.1. Cloning, Protein Expression, and Purification

Construction of AR N-terminal domains: Fragments encompassing 30 amino acids on each side preceding and following the polyQ stretches of 22 and 45 from ARQ22 and ARQ45 were obtained by PCR amplification of the respective regions from wild-type or mutant AR expression vectors with *Bam* HI and *Eco* RI sites and ligated into the corresponding sites in pGEX-6T-1 expression vector (Addgene, Cambridge, MA, USA). The fragments cloned are as follows: AR (90–270), ARQ22 (32–270), and ARQ45 (32–293). Each of these fragments was cloned with either the wild-type KELCKAVSVSM (237–247) motif or the KELSKAVSVSM motif with a mutated cysteine. The constructs were transformed into BL21 (DE3) *E. coli*, and the proteins were isolated on glutathione sephadex beads. The same six AR-NTD sequences were cloned into the *Bam* HI and *Eco* RI sites of the expression vector pET-GB1a and transformed into BL21 (DE3) *E. coli* for protein production. The resulting proteins were immobilized on Ni–agarose beads. The beads were washed extensively with 50 mM NaH_2_PO_4_, 300 mM NaCl, and 20 mM imidazol. Thereafter, the proteins were eluted with 50 mM NaH_2_PO_4_, 300 mM NaCl, and 250 mM imidazole, dialysed, and concentrated using Amicon Ultra-15 Centrifugal Filter Units (Merck Millipore, Dachstein, France). Tobacco etch virus (TEV) protease was added and incubated at 4 °C for 30 min with continuous rotation to cleave off the beta 1 immunoglobulin binding domain of protein G GB1a tag. After the cleavage, the AR-NTD proteins were separated from the His-tag by capturing the GB1 His tag on a Ni–agarose matrix to recover the free non-tagged proteins.

SUMO-fusion constructs: The KELCKAVSVSM sequence was first cloned into a pETHis1a SUMO expression vector. Using the expression vector as a PCR template, a PCR reaction was performed with a T7 forward primer, 5′-TAATACGACTCACTATAGGGGAATTGTG-3′, and a reverse primer, 5′GGATCCTCACATGGACACCGACACTGCCTTACACAACTCCTTTGGCGCAGATCCACCAATCTGTTCCTGTGAGC-3′. The reverse primer carried a *Bam* HI restriction site, a stop codon, the AR peptide sequence KELCKAVSVSM, and the C-terminal part of the SUMO protein (excluding the enhanced green fluorescent protein). The reverse primer also coded for a three amino acids Ser-Ala-Pro linker that was inserted between the peptide and the SUMO tag. An Xba I restriction site located downstream of the T7 promoter in the expression vector and a *Bam* HI restriction site were used for the cloning to generate SUMO_SAPKELCKAVSVSM construct that was verified by DNA sequencing.

*E. coli* Rosetta (DE3) competent cells were used as the host strain for protein expression. The cells were transformed with the construct and protein expression induced either in Luria broth (LB) containing ^15^NH_4_Cl (0.5 g) and ^13^C uniformly labeled glucose (2 g) per liter of culture as the sole source of nitrogen and carbon, respectively, for NMR analysis [27,28]. The cells were harvested in a lysis buffer (50 mM Tris-HCl, pH 8.0; 150 mM NaCl; 10 mM imidazole; 2 mM β-mecaptoethanol; 0.2% NP-40; 2.5 U/mL DNase 1; 2.5 mu/mL RNase A) and a tablet of ethylenediaminetetraacetate (EDTA)-free protease inhibitor cocktail (Roche), and six sonication steps of 1 min each were performed with a Branson digital sonicator. The total cell lysate was centrifuged at 36,000 rpm for 1 h at 4 °C and the soluble fraction filtered through a Minisart High Flow syringe filter (Sartorius Biotech, Goettingen, Germany) with a pore size of 0.20 μm. The supernatant was loaded onto a Ni–NTA agarose resin column, pre-equilibrated with the lysis buffer for gravity-flow chromatography using Econo-Pac columns (Bio-rad, Marnes-la-Coquette France). The resin-bound protein was washed successively with the lysis buffer, Wash Buffer 2 (the lysis buffer minus NP-40), Wash Buffer 3 (Wash Buffer 2 plus 1 M NaCl), Wash Buffer 4 (Wash Buffer 2 plus 20 mM imidazole), and finally eluted with the Wash Buffer 2 containing 330 mM imidazole and 10% glycerol.

### 4.2. Size Exclusion Chromatography Analysis of SUMO–Peptide Fusion

The affinity purified SUMO-SAPKELCKAV fusion peptides were concentrated and analyzed by size exclusion chromatography by injecting the samples onto a HiLoad 16/60 Superdex 75 prep grade column (GE Healthcare, Buc, France) and pre-equilibrated with 20 mM sodium phosphate buffer (pH 6.5) and 150 mM NaCl, with or without 2 mM DTT.

### 4.3. NMR Experiments and Formation of Amyloid Fibrils of KELCKAV Peptides

The KELCKAV peptide in the SUMO construct was cleaved from the SUMO tag by incubating the fusion protein with an “in-house” produced SUMO protease at a ratio of 1:100 for 1 h at 30 °C. The cleaved peptide was then purified by reverse-phase high-pressure liquid chromatography (RP-HPLC) using a preparative scale C18 column (PrePak cartridge Waters, Guyancourt, France, 21 × 250 mm, 300 A, 5 μM) with an acetonitrile gradient ranging from 10 to 70% in 0.1% trifluoracetic (TFA). The peptide fractions were pooled together, lyophilized, and either used immediately or stored at −20 °C for later use. All NMR measurements were recorded on a 700 MHz Bruker Avance III HD spectrometer equipped with a Z gradient triple resonance cryogenic probe. Resonance assignments of the RP-HPLC purified KELCKAVSVSM peptides were obtained using standard homonuclear proton spectra (TOCSY and NOESY), ^1^H-^15^N- and ^1^H-^13^C HSQC heteronuclear correlation spectra and HNCA triple resonance spectra recorded at 298 K [29]. Proton chemical shifts were referenced using the 2,2-dimethyl-2-silapentene-5-sulfonate (DSS) as an external standard, while ^15^N and ^13^C chemical shifts were calibrated indirectly using the values of their magnetogyric ratios [30]. As a control, ^1^H-^15^N HSQC heteronuclear correlation spectra for the SUMO–peptide fusion were also recorded. All spectra were processed using Topspin 2.1 (Bruker, Rheinstetten, Germany) and analyzed using CcpNmr [31].

The formation of KELCKAVSVSM fibrils was performed as previously described [16]. Peptide fractions from RP-HPLC were pooled and lyophilized to remove acetonitrile. TFA was then removed from the peptide samples by three rounds of acidification (2 mM HCl) and lyophilization as described by Andrushchenko et al. [32]. The peptide was then resuspended in 100% D_2_O, the pH was adjusted to 7.0, and 10% DMSO was added to produce a final volume of 150 μL at a 660 μM peptide concentration. The kinetics of fibril formation was monitored by NMR at 25 °C and the fibrils imaged by transmission electron microscopy.

### 4.4. AFM Measurements of ARNTD Constructs

The affinity purified AR-NTD proteins were incubated at 60 μM in 50 mM Tris-HCl, pH 8.0 for 18 h at 37 °C to initiate the aggregation process before spotting on mica for AFM measurements. The samples were measured using a Nanoscope Dimension ICON (Bruker, Rheinstetten, Germany) in tapping mode in air with a scan rate of 1 Hz.
